# CD8+ T Lymphocytes Immune Depletion and LAG-3 Overexpression in Hodgkin Lymphoma Tumor Microenvironment Exposed to Anti-PD-1 Immunotherapy

**DOI:** 10.3390/cancers13215487

**Published:** 2021-10-31

**Authors:** Jean-Marie Michot, Severine Mouraud, Julien Adam, Julien Lazarovici, Camille Bigenwald, Charlotte Rigaud, Lambros Tselikas, Peggy Dartigues, Alina Danu, Amélie Bigorgne, Veronique Minard, David Ghez, Aurélien Marabelle, Laurence Zitvogel, Vincent Ribrag

**Affiliations:** 1 Gustave Roussy, Département des Innovations Thérapeutiques et Essais Précoces, Université Paris-Saclay, F-94805 Villejuif, France; AURELIEN.MARABELLE@gustaveroussy.fr (A.M.); vincent.ribrag@gustaveroussy.fr (V.R.); 2 Gustave Roussy, Inserm U1015, Université Paris-Saclay, F-94805 Villejuif, France; severine.mouraud@gustaveroussy.fr (S.M.); amelie.bigorgne@gustaveroussy.fr (A.B.); Laurence.zitvogel@gustaveroussy.fr (L.Z.); 3 Gustave Roussy, Department of Biopathology, Université Paris-Saclay, F-94805 Villejuif, France; jadam@ghpsj.fr (J.A.); peggy.dartigues@gustaveroussy.fr (P.D.); 4 Gustave Roussy, Departement of Hematology, Université Paris-Saclay, F-94805 Villejuif, France; julien.lazarovici@gustaveroussy.fr (J.L.); camille.bigenwald@gustaveroussy.fr (C.B.); Alina.danu@gustaveroussy.fr (A.D.); david.ghez@gustaveroussy.fr (D.G.); 5 Gustave Roussy, Department of Pediatric and Adolescent Oncology, Université Paris-Saclay, F-94805 Villejuif, France; charlotte.rigaud@gustaveroussy.fr (C.R.); Veronique.MINARD@gustaveroussy.fr (V.M.); 6 Gustave Roussy, Departement of Interventional Radiology, Université Paris-Saclay, F-94805 Villejuif, France; lambros.tselikas@gustaveroussy.fr

**Keywords:** Hodgkin lymphoma, immune checkpoint inhibitors, LAG-3, anti-PD-1

## Abstract

**Simple Summary:**

Immune checkpoint blockers are important immunotherapies for the treatment of patients with Hodgkin lymphoma. The resistance mechanisms of these immunotherapies remain unknown. This pilot study aims to decipher the resistance mechanisms of immunotherapies in Hodgkin lymphoma. The main results show that immunotherapy-resistant Hodgkin lymphoma had CD8 lymphocytes depleted in microenvironment and overexpression of the LAG-3 molecule. This study proposes hypotheses for understanding the resistance to immunotherapies in patients with Hodgkin lymphoma.

**Abstract:**

*Background*: Resistance to anti-PD-1 remains a considerable clinical challenge for the treatment of patients with classical Hodgkin lymphoma (cHL), and mechanisms of anti-PD-1 resistance remain unknown. This pilot study aims to investigate the tumor microenvironment in patients with cHL relapsing after anti-PD-1. *Methods*: This study investigated tumor samples of eight patients with cHL, including four patients exposed to anti-PD-1 with a paired longitudinal histological analysis before and after anti-PD-1, and four patients not exposed to anti-PD-1 who served as control for the cellular biological investigations. Fresh cells tumor microenvironment analysis included phenotypic characterization of their T cell surfaces immune checkpoint markers PD-1, PD-L1, ICOS, TIM-3, LAG-3, 41-BB and BTLA. Tumor tissues immunohistochemistry staining included CD30, CD4, CD8, CD68, CD163, PD-L1, PD-1, LAG-3 and TIM-3. *Findings*: Paired longitudinal tumor tissues analysis in the tumor microenvironment found a CD8+ lymphocytes tumor depletion and an increase of LAG-3 staining after anti-PD-1 exposure. The fresh cells analysis of the tumor microenvironment in patients exposed to anti-PD-1 found CD8+ lymphocyte depletion, with an elevated CD4+/CD8+ lymphocytes ratio (median ratio 9.77 in exposed anti-PD-1 versus 2.39 in not-exposed anti-PD-1 patients; *p* = 0.0943). On the cell surfaces of CD4+ lymphocytes, the median positive expression of LAG-3 was significantly higher in the samples exposed to anti-PD-1 compared to the controls (15.05 [IQR:17.91–10.65] versus 3.84 [IQR 1.87–6.57]; *p* = 0.0376). *Interpretation*: This pilot study proposes hypotheses for understanding the resistance to immunotherapies in patients with Hodgkin lymphoma. Hodgkin lymphoma exposed to anti-PD-1 correlated in tumor microenvironment with an immune depletion of CD8+ T lymphocytes and overexpression of LAG-3 on CD4+ helper T lymphocytes.

## 1. Introduction

Anti-PD-1 has shown significant and long-lasting efficacy in the treatment of relapsed classical Hodgkin lymphoma (cHL) after chemotherapy [1,2,3]. The response rates of anti-PD-1 in patients with relapsed or refractory cHL range from 69% to 72% of overall response, including 16% to 28% of complete responses [2,3]. However, resistance to anti-PD-1 remains a problem in the management of patients with Hodgkin lymphoma [4,5]. Research efforts into understanding the Hodgkin lymphoma disease and drug resistance must continue to improve the effectiveness of treatments. Investigating the mechanisms of resistance to anti-PD-1 in cHL could further lead to optimizing the next therapeutic strategies.

Hodgkin lymphoma is one of the most sensitive tumor types to anti-PD-1 inhibition, and this tumor is characterized by an ineffective but rich tumor microenvironment [6,7]. Chromosome 9p24.1 alterations are a defining feature of classical Hodgkin lymphoma, resulting in PD-L1 and PD-L2 overexpression [8]. Our understanding of immunological changes within the Hodgkin tumor microenvironment during or after chemotherapy or anti-PD-1 immunotherapy remains limited. Taylor and al. found programmed cell death protein-1 (PD-1) expression and its ligand PD-L1 in the microenvironment of classical Hodgkin lymphoma does not enrich over serial relapses with conventional chemotherapy [9]. Sasse and al. reported a patient case series that illustrated that, at relapse after anti-PD-1, the target PD-1 was present on T-cells in the vicinity of Reed Sternerg cells, at a level easily detectable by immunohistochemistry [10]. The Sasse and al. results suggested that therapeutic resistance to anti-PD-1 could bypass the PD-1-PD-L1 axis, potentially by alternative immune checkpoints. We propose to investigate the tumor microenvironment biological mechanisms of resistance to anti-PD-1 in tumor lymph nodes of patients with relapsed or refractory Hodgkin lymphoma exposed to anti-PD-1.

## 2. Material and Methods

### 2.1. Patients

**Figure 1:**
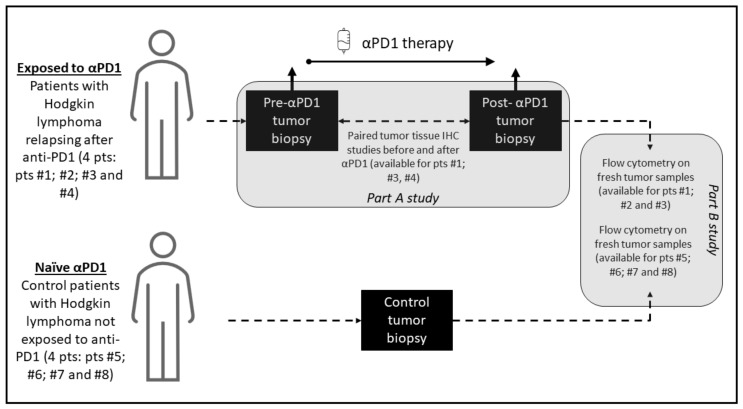
Graphical summary of the study methodology. The study consisted of two parts: part A, which was a paired study of tumor biopsies before and after anti-PD-1 exposure where each patient is his own control, and part B, which compared analyses on fresh cells of tumor microenvironment of patients exposed to anti-PD-1 and not exposed to anti-PD-1.

The study investigated 8 patients with cHL; including 4 patients exposed to anti-PD-1 with a paired longitudinal histological analysis before and after anti-PD-1, and 4 patients not exposed to anti-PD-1 who served as control for the cellular fresh cells analysis of the tumor microenvironment tumor, including phenotypic characterization of T cell and immune checkpoint markers PD-1, PD-L1, ICOS, TIM-3, LAG-3, 41-BB and BTLA. Tumor biopsy immunohistochemistry staining was performed before anti-PD-1 and after PD-1 exposure and included CD30, CD4, CD8, CD68, CD163, PD-L1, PD-1, lymphocyte-activation gene 3 (LAG-3) and T-cell immunoglobulin and mucin-domain containing 3 (TIM-3). The study included patients treated for Hodgkin lymphoma at Gustave Roussy, Villejuif, France, between October 2016 and December 2018. The Hodgkin lymphoma pathological subtypes classification was done according to WHO 2016 classification of lymphoid neoplasms [11]. The criteria for the longitudinal histological analysis before and after anti-PD-1 included patients with a tumor progression confirmed on a computerized tomography scan following anti-PD-1 therapy, with enough archivable tumor material available for the study. The objective of the study was to describe the cellular and microenvironment tumor tissue modifications after exposure to anti-PD-1 in cHL. The biological studies consisted of two parts: parts A for tumor tissue paired analysis and part B for fresh cells analysis. A graphic summary of the study methodology and investigations performed is presented in Figure 1.

Part A of the study consisted of tumor tissues longitudinal anatomopathological investigations on paired tumor biopsies; before and after anti-PD-1 of the same patient. In this part of the study, archived tumor material was required, for a standardized and centralized comparative analysis of the paired tumor biopsies. Analysis of paired biopsies investigated the immunohistochemical changes after anti-PD-1 immunotherapy. For each immunochemistry staining, a quantification technique was used with a cell density staining study per mm^2^. The tumor samples were reviewed by two expert pathologists (PD and JA) to validate the cHL diagnosis, evaluate the amount of tumor tissue present in hematoxylin and eosine-stained slides, and select appropriate blocks. For patients with paired biopsies available, formalin-fixed, paraffin-embedded tissue samples were analyzed for markers CD30, PD-1, PD-L1, LAG-3, TIM-3, CD4, CD8, CD68 and CD163. Representative tumor samples were analyzed by immunohistochemistry to determine the expression of CD30 (clone Ber-H2, Agilent Technologies, Santa Clara, CA, USA), PD-1 (clone NAT105; Abcam, Cambridge, UK), PD-L1 (clone E1L3N; Cell Signaling Technology, Danvers, MA, USA), LAG-3 (clone D2G40; Cell Signaling Technology, Danvers, MA, USA), TIM-3 (clone D5D5R; Cell Signaling Technology, Danvers, MA, USA), CD4 (clone SP35; Roche Pharma, Basel, Suisse), CD8 (clone C8/144B; Agilent Technologies, Santa Clara, CA, USA), CD68 and CD163 (clone PGM1, Agilent Technologies Santa Clara, CA, USA and clone 10D6 Diagnostic BioSystems, Pleasanton, CA, USA, respectively), as previously described [12]. Staining was performed on 4 mm formalin fixed, paraffin-embedded tissue sections using a Ventana Benchmark platform (Ventana, Oro Valley, AZ, USA). Hodgkin Reed Sternerg cells were identified morphologically at microscopic examination of the stained slides. Hodgkin Reed Sternerg cells and microenvironment cells were differentiated by means of CD30 staining and by standardized characterization of the distinct morphology of the RS-cells and macrophage nuclei. An assessment of cell densities (number of cells per square mm) was performed by immunohistochemical staining image analysis for the studied markers (CD4, CD8, LAG-3, TIM-3, PD-L1, PD-1), before and after anti-PD-1 immunotherapy.

Part B of the study consisted of a flow cytometry of fresh cells from the tumoral microenvironment. In this part of the study, patients with cHL who did not receive anti-PD-1 immunotherapy were control patients. Fresh biopsies of lymph nodes from cHL patients were manually crushed with the bottom of a syringe and 1X PBS (Phosphate Buffer Staining) over a 70 μm cell strainer (BD Biosciences, Franklin Lakes, NJ, USA). Cell suspensions were washed and resuspended in 1X PBS and stained for flow cytometry analysis. The phenotypical analysis of T cells in the microenvironment of lymph node biopsy was performed as follows. To evaluate the number of immune cells, a kit (Precision Counts Beads^TM^, Biolegend, San Diego, CA, USA) was used, according to the manufacturer’s instructions. Between 100,000 and 300,000 CD45+ cells were stained for each panel. Cells were incubated with LIVE/DEAD™ Fixable Yellow Dead Cell Stain Kit (Invitrogen, Waltham, Mass. USA), and a combination of human antibodies was applied including anti-CD45 BUV805 (HI30), anti-CD3 BUV395 (SK7), anti-CD4 BUV496 (SK3), anti-CD19 PeCy7 (SJ25C1), anti-TIM-3 BV650 (7D3), anti-ICOS PerCpCy5.5 (DX29), anti-CD45Ra BV650 (HI100), anti-CCR7 APC-R700 (3D12), anti-CCR6 AlexaFluor 647 (11A9), anti-CXCR3 PeCy7 (1C6/CXCR3), anti-CRTH2 FITC (BM16) from Becton Dickinson^®^, Franklin Lakes, NJ, USA; anti-CCR10 PE (314305) from R&D Systems^®^, Minneapolis, MN, USA; anti-PD-1 PE (MIH4), anti-LAG-3 FITC (3DS223H), anti-FoxP3 APC (PCH101) from eBiosciences^®^, San Diego, CA, USA; anti-CD25 PeCy7 (A52882) and anti-CD56 PeCy7 (N901) from Beckman Coulter^®^, Brea, CA, USA; anti-PD-L1 APC (29E2A3), anti-CD8 PerCpCy5.5 (SK1), anti-41BB PECF594 (4B4-1), anti-BTLA APC (MIH26), from Biolegend^®^ San Diego, CA; and finally anti-CD8 FITC (REA734) from Miltenyi^®^; Bergisch Gladbach, Germany. All surface staining was performed at +4 °C for 15 min. The cells were then washed two times and were acquired on an 18-colors flow cytometer BD Fortessa (BD Biosciences). Data were acquired in FCS 3.0 format and analyzed with a KALUZA software version 2.1.

### 2.2. Statistical Analysis and Ethics

Data were quoted as the median (range), and for histogram analysis the interquartile range was shown. The mean fluorescence intensity (MFI) was measured by flow cytometry, after adding the revelation reagent investigated. A nonparametric Mann–Whitney test and an unpaired *t*-test with Welch’s correction were used to compare biologicals quantitative variables. The threshold for statistical significance was set to *p* < 0.05. All statistical analyses were performed using GraphPad Prism software (version 8.1.2 GraphPad Software Inc., San Diego, CA, USA).

The study was approved by the local institutional review board and hematological committee and the research steering committee of Gustave Roussy. The study carried out complies with articles of French Law Number° L1211-2 and follows rules of the public health code. All patients gave written informed consent for authorization for the use, for research purposes, of the remains of samples taken for diagnostic and/or therapeutic purposes. This study was conducted in accordance with the Declaration of Helsinki.

### 2.3. Role of the Funding Source

This study was funded by Gustave Roussy as part of the Gustave Roussy Immunotherapy Program (GRIP, launched in 2015), a dedicated institutional program designed to accelerate the clinical development of immunotherapy and strengthen translational research. The funding source had no influence on the data collection or the presentation of the results. SM, JA, JMM had full access to the raw data. All authors approved the final manuscript. The corresponding author had full access to the data in the study, and the final responsibility and decision to submit the manuscript for publication.

## 3. Results

### 3.1. Characteristics of Patients

Eight patients were included in the study, four patients were exposed to anti-PD-1 and relapsed, and four patients were not exposed to anti-PD-1 who served as control patients. The detailed clinical characteristics of the eight patients (five male and three female) are shown in Table 1 and Table 2. Median (range) age was 41.5 (16–76) years old. Patients treated with anti-PD-1 received pembrolizumab 200 mg intravenously, every 3 weeks for up to 2 years of therapy.

### 3.2. Paired Tumor Tissue Analysis before and after Anti-PD-1 (Part A Results)

**Figure 2:**
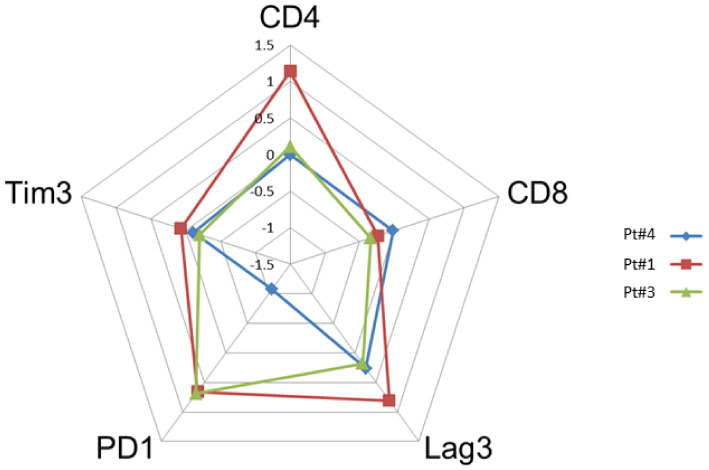
Comparison of the intensity of staining by immunohistochemistry of tumor biopsies before and after anti-PD-1 (paired biopsy analysis, part A study). The evaluation of the cell densities (number of cells per square mm) was performed by immunohistochemical staining image analysis for the CD4, CD8, Lag-3, Tim3 and PD-1 markers in patients #1, #3 and #4 before and after anti-PD-1 treatment. The diagram shows a summary of the fold changes for the main relevant markers. The numeric scale shown is 10 logs (logarithmic scale) and indicates the variations before and after anti-PD-1 for each marker studied. 0 indicates no change, 0.5 indicates a 2-fold increase in staining, and 1 indicates a 10-fold increase in the staining studied.

**Figure 3:**
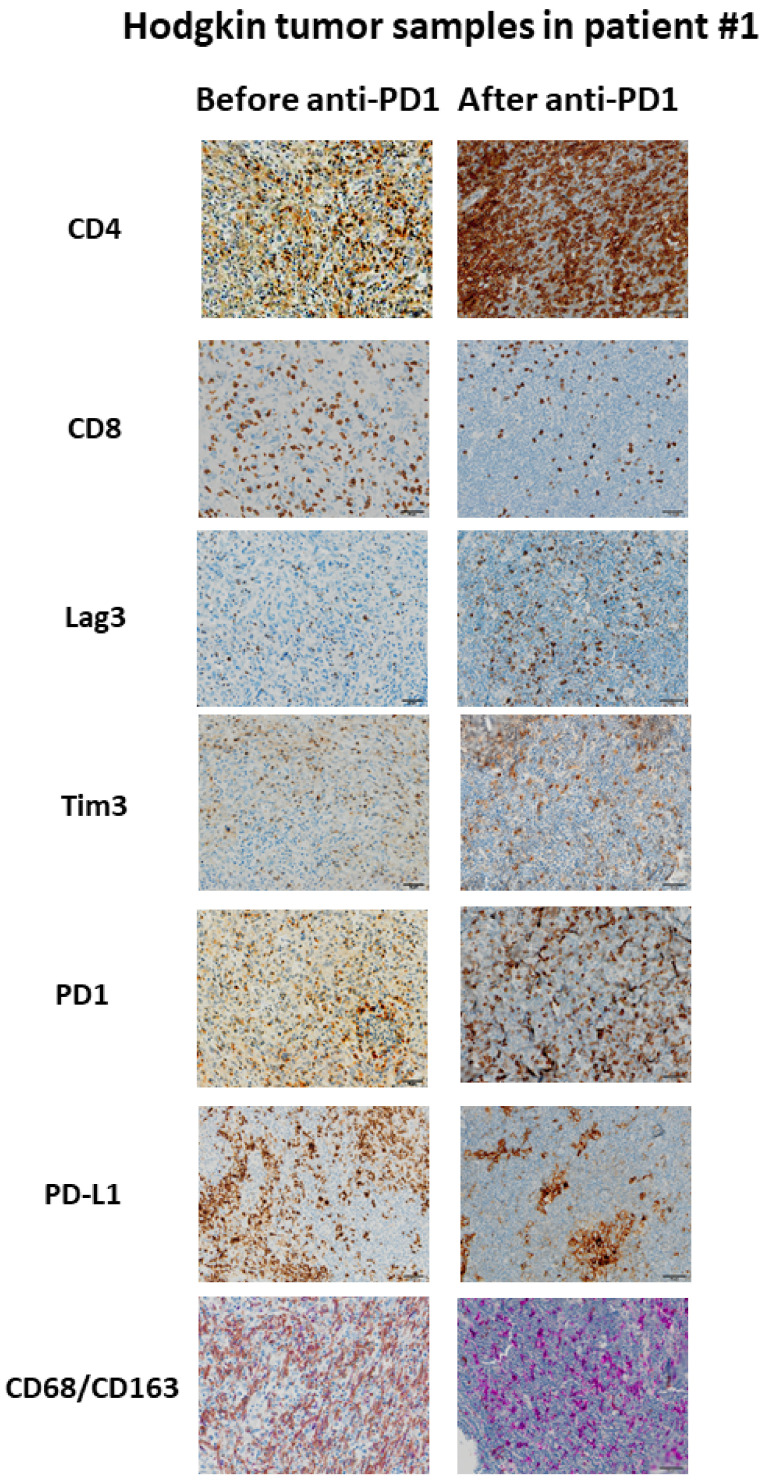
Example of immunological changes before and after anti-PD-1 on paired tumor biopsies (paired biopsy analysis, part A study). Pictures were captured from tumor samples of paired tumor biopsy before and after anti-PD-1 immunotherapy from patient #1, with immunohistochemistry staining for CD4, CD8, LAG-3, Tim3, PD-1, PD-L1, CD68/CD163. The scale bar on each image (lower right corner) is equal to 50 μm.

**Figure 4:**
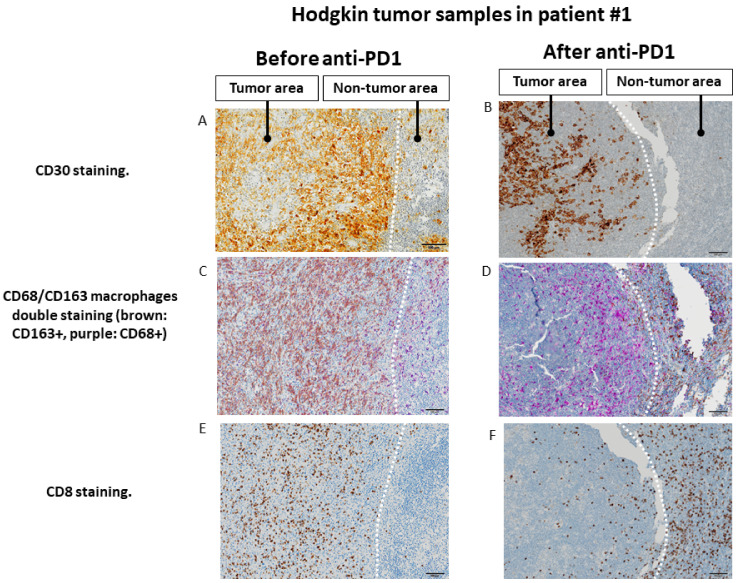
Changes in the spatial tumor distribution of CD8 lymphocytes and CD68/CD163 macrophages before and after anti-PD-1 immunotherapy by immunohistochemistry in samples from patient #1 (fresh tumor microenvironment cells analysis, part B study). (**A**,**B**): CD30 immunohistochemical staining showing tumor areas with CD30+ Reed Sternberg of Hodgkin cells, in contrast to residual adjacent non-tumor lymphoid tissue. (**C**,**D**). Before exposure to anti-PD-1, tumor area is infiltrated by predominantly CD163+ macrophages, whereas adjacent lymphoid tissue is infiltrated mainly by CD68+CD163− cells. After exposure to anti-PD-1, macrophages in the tumor area show only a CD68+CD163− phenotype, whereas CD163 macrophages seem to be excluded from the tumor zone. (**E**,**F**). Before exposure to anti-PD-1, the tumor zone is rich in CD8+ T lymphocytes. After anti-PD-1, the tumor area is depleted of CD8+ cells while the adjacent lymphoid tissue appears rich in CD8+ T lymphocytes. The scale bar on each image (lower right corner) is equal to 100 μm.

The paired tumor tissues biopsies investigations were performed before and after anti-PD-1, in the same patient for comparisons of immunochemistry staining intensity analysis in tumor microenvironment. Sufficient tumor materials for all biomarkers were available for pts #1, #3 and #4. After anti-PD-1 exposure, PD-1 remained positively expressed with more than a 2-fold higher expression compared with pre-anti-PD-1 biopsy in pts #1 and #3. PD-1 staining intensity decreased after anti-PD-1 exposure in samples of pt #4 (Figure 2). After anti-PD-1 exposure, CD8 staining intensity decreased in two patients (pts #1 and #3) and remained stable in patient #4. CD4 staining intensity was stable in two patients (pts #3 and pt #4) and increased distinctly in pt #1 after anti-PD-1 exposure (16-fold increase; log_10_ (16) = 1.2 on the logarithmic scale). The LAG-3 staining intensity increased after exposure to anti-PD-1 in three patients tested on the paired biopsies. We observed patient #1 had the most distinct LAG-3 staining intensity increase at 7-fold higher after anti-PD-1 compared to before anti-PD-1 (log_10_ (7) = 0.8 on the logarithmic scale) (Figure 3). Overall, after anti-PD-1 exposure, the distribution of immune cells in the tumor microenvironment was predominantly composed of CD4+ T lymphocytes and was generally poor in CD8+ T cells (Figure 2 and Figure 3). Large samples from adenectomy post-anti-PD-1 were available for pt #1, showing a near complete spatial tumor exclusion of T CD8+ lymphocytes and macrophages CD163+ (Figure 4). In patient #1, after exposure to anti-PD-1, nodal tissue tumor spatial analysis found CD8+ lymphocytes were almost exclusively on the periphery of the tumor zone, and macrophages at the periphery of the tumor area were a mixture of CD68+ and CD163+.

### 3.3. Fresh Cells Tumor Microenvironment Analysis by Flow Cytometry (Part B Results)

**Figure 5:**
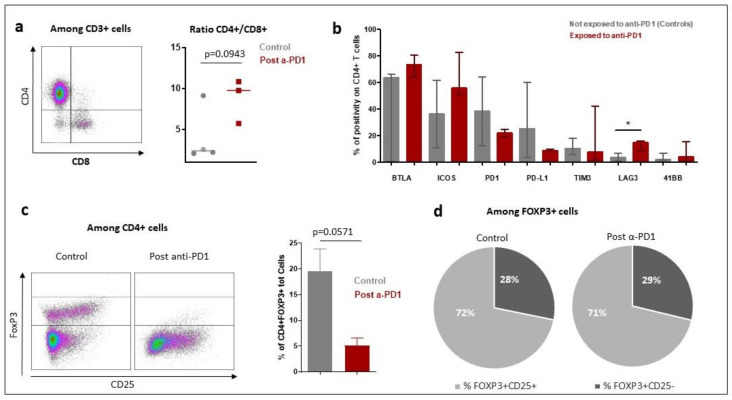
Characteristics of tumor microenvironment CD4+ T-cell population in patients with Hodgkin lymphoma exposed to anti-PD-1 and naïve to anti-PD-1 (control patients) by fresh tumor microenvironment cells analysis (part B study). The characteristics of the CD4+ lymphocytes in the tumor microenvironment were compared between patients who received immunotherapy (post α-PD-1 patients; *n* = 3) and controls patients not exposed to anti-PD-1 (control patients; *n* = 4). (**a**): Overview of CD4+ and CD8+ T lymphocytes distribution in patients exposed to anti-PD-1 and control patients not exposed to anti-PD-1. The bar indicates the median of the values. (1) *p*-value was determined with a Mann–Whitney test with Welch’s correction. (**b**): Expression of immune checkpoint markers on CD4+ T cell surfaces of tumor microenvironment. The rectangle indicates the median of values and the bar the interquartile range. *p*-value was determined with a Mann–Whitney test and Welch’s correction. * Indicates the median positive expression of LAG-3 was found significantly higher in exposed to anti-PD-1 samples compared to controls (15.05 [IQR:17.91–10.65] versus 3.84 [IQR 1.87–6.57]; *p* = 0.0376). Other markers did not significantly vary between controls and exposed to anti-PD-1 samples. (**c**): Proportion of FoxP3+ Tregs cells among CD4+ T-cells. In the left part of the figure are two representative dot plots showing FoxP3+ T regulatory cells, one from a control patient and another from post-anti-PD-1 treated patient. On the right part of the figure is shown the bars chart of relative proportion of CD4+ FoxP3+ regulatory T cells. *p*-value was determined with a Mann–Whitney test and Welch’s correction. (**d**): Distribution of activated and non-activated regulatory T cells in tumor microenvironment.

**Figure 6:**
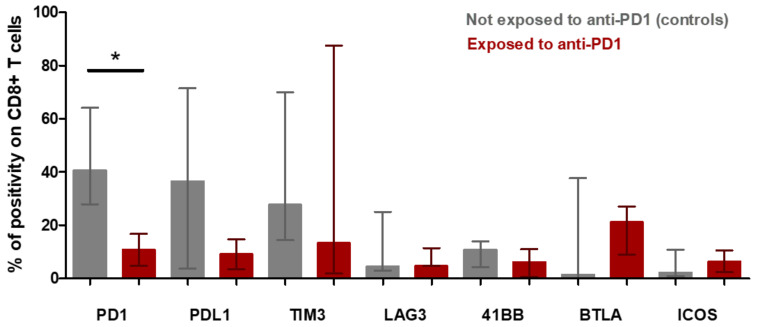
CD8+ t-cell population in microenvironment of Hodgkin lymphoma from patients exposed to anti-PD-1 and not exposed to anti-PD-1 (control patients) (fresh tumor microenvironment cells analysis, part B study). Expression of immune checkpoint markers on CD8+ T cell surfaces of tumor microenvironment. The rectangle indicates the median of values and the bar the interquartile range. The *p*-value was determined with a Mann–Whitney test with Welch’s correction. * Indicates the median positive expression of PD-1 was found significantly lower in exposed to anti-PD-1 samples compared with controls (10.82 [IQR:5.21–18.30] versus 40.67 [IQR 27.69–64.04]; *p* = 0.0431). Other markers did not significantly vary between controls and exposed to anti-PD-1 samples.

The tumor microenvironment cell fresh flow analysis was available for seven patients: three patients exposed to anti-PD-1 (Pts #1, #2, #3) and four control patients not exposed to anti-PD-1 (pts #5, #6, #7 and #8). The median percentage of CD45^+^ immune cell infiltration in tumor microenvironment was 91% in patients having relapsed after anti-PD-1 and 57% in control patients (*p* = 0.1024) (Figure S1). The median of CD4+/CD8+ T-cells ratio was 9.77 in patients exposed to anti-PD-1 and 2.39 in control patients (*p* = 0.0943) (Figure 5a). To further determine the phenotype of the CD4^+^ T cell subset, a multicolor flow cytometric determination of T lymphocytes on fresh cells was performed. There was a trend towards a lower proportion of CD4+FoxP3+ regulatory T-cells in the tumor microenvironment of patients exposed to anti-PD-1 compared to control patients (*p* = 0.0571; Figure 5c). Among FoxP3+ cells, the proportion of activated FoxP3+CD25+ and non-activated FoxP3+CD25− cells was similar in exposed anti-PD-1 patients and control patients (Figure 5d). Cell surface expression of PD-1, PDL-1, TIM-3, LAG-3, 41BB, BTLA and ICOS immune checkpoints markers on CD4+ and CD8+ T-cells were investigated. In the CD4+ lymphocytes surface cells, the median positive expression of LAG-3 was significantly higher in those exposed to anti-PD-1 samples compared to control patients (15.05 [IQR:17.91–10.65] versus 3.84 [IQR 1.87–6.57]; *p* = 0.0376) (Figure 5b). In the CD8+ lymphocytes surface cells, the median positive expression of PD-1 was significantly lower in those exposed to anti-PD-1 samples compared to control patients (10.82 [IQR:5.21–18.30] versus 40.67 [IQR 27.69–64.04]; *p* = 0.0431) (Figure 6).

## 4. Discussion

Our study investigates the changes in tumor microenvironment in patients with classical Hodgkin lymphoma after anti-PD-1 exposure. By combining flow cytometry and tumor tissues immunohistochemistry techniques, four biological features were reported after anti-PD-1 exposure in a tumor microenvironment of Hodgkin lymphoma.

The first finding was depletion of CD8+ T lymphocytes in the tumor microenvironment of anti-PD-1-exposed samples in Hodgkin lymphomas. One patient (pt #1) with large adenectomy provided a comprehensive architectural tumor tissue analysis and had a nearly complete exclusion of CD8+ lymphocytes outside of the tumor area after anti-PD-1 therapy. In this patient, after anti-PD-1 exposure, the tumor microenvironment appeared predominantly composed with CD4+ helper T lymphocytes. The fresh cells analysis confirmed this observation and found CD8+ T lymphocytes depletion with ratio CD4+/CD8+ elevated after anti-PD-1 exposure as compared with controls. The regulatory T cell population tended to be lower in patients relapsed after anti-PD-1, compared to control patients, as CD4+ lymphocyte population does not overexpress FOXP3+. The sum of these data suggests that the CD4+ cell population found in anti-PD-1 exposed tumors samples, does not belong to the regulatory T population. Further studies including in-depth functional tests of CD4+ remain warranted to characterize this CD4+ population implicated in resistance to anti-PD-1.

The second finding in our study was an overexpression of LAG-3 on the CD4+ lymphocyte surfaces of the microenvironment of Hodgkin lymphoma, in patients exposed to anti-PD-1 immunotherapy. LAG-3 is highly structurally homologous to CD4 with four extracellular immunoglobulin superfamily-like domains, and is transmembrane protein identified on activated human T cells and NK cells [13,14,15]. In addition, the mice model revealed that a fibrinogen-like protein 1 (FGL1), a liver-secreted protein, is a major LAG-3 functional ligand independent from MHC-II [16]. LAG-3 is an immune checkpoint thought to be associated with immunological tumor escape in several types of solid tumors [17,18,19,20,21] as well as in Hodgkin lymphoma [4,22,23,24]. LAG-3 was found expressed on a majority of tumor infiltrating lymphocytes in pediatric Hodgkin lymphoma, and there was a positive relationship between the presence of LAG-3 and PD-L1 expression [25]. A pre-clinical study reported that MHC class II engagement by its ligand LAG-3 contributes to melanoma resistance to apoptosis [17]. Other researchers found a striking synergy between LAG3 and PD-1 inhibitory pathways and argue that dual blockade of these molecules represents a promising combinatorial strategy to treat cancer [26]. Interestingly, recent results of a phase 3 clinical trial in patients with metastatic melanoma showed that anti-LAG-3 antibody combined with anti-PD-1 improves overall survival in patients with metastatic melanoma, compared with anti-PD-1 monotherapy [27].

The third finding in our study was that anti-PD-1 staining on the tumor microenvironment generally remained positive, even though patients had recently been treated with anti-PD-1 immunotherapy. This result was consistent with the results of Sasse and al. [10], who found that in the tumor microenvironment of cHL, patients relapsed after anti-PD-1; PD-1 were decreased but remained positively expressed on T cell surfaces at a level easily detectable by immunohistochemistry. In our study with fresh cells analysis, we did not find changes of PD-1 staining on the CD4+ T lymphocytes of tumor microenvironment, whether patients were exposed or naïve to anti-PD-1. In contrast, we observed a decrease in PD-1 staining on the surface of CD8+ T lymphocytes in patients exposed to anti-PD-1 (Figure 6). These results suggest that the therapeutic target of the PD-1-PD-L1 axis remains probably engaged in the intratumoral CD8 lymphocytes following anti-PD-1 treatment, and that the mechanisms of therapeutic resistance bypass the PD-1-PD-L1 axis and would then go through other checkpoint inhibitors molecules.

The last finding in our study was observation in one patient (pt #1) providing large adenectomy of spatial changes in distribution of immune cells within the tumoral lymph node post-exposure to anti-PD-1. A spatial immunological exclusion of CD8+ T lymphocytes and CD163+ macrophages outside the tumor areas was found after anti-PD-1 in this patient #1. This observation could argue in favor of a polarization of macrophages to M1-like profiles (CD68+) within the tumor [28], after exposure to anti-PD-1 immunotherapy. However, this phenomenon of spatial immune exclusion was observed in only one patient in our study and needs to be confirmed. Other researchers investigating resistance to anti-PD-1 in other tumor types (melanoma) with paired tumor biopsy found similar results of immunological exclusion phenomenon, with immunohistochemical staining abundant CD8 T-cell infiltrates at baseline and at the time of relapse only restricted at the tumor margin [29]. It was also recently reported, in a paired biopsy analysis, that CD8+ T cells and immunosuppressive markers were all reduced after anti-PD-1 in a patient with squamous cell carcinoma of the head and neck [30]. Precise biological processes that lead to such immune spatial exclusion phenomenon remain to be further elucidated.

The main limitation in our study was the small number of patients included, due to difficulties in obtaining tumor sample sizes large enough to perform all the expected investigations. This limit leads to consider the interpretation of the results with caution and to study more forward the role of LAG-3 and spatial changes of CD8 in relapsing tumors after anti-PD-1. Secondly, further analysis such as in-depth cellular, immunological or tumoral molecular investigations could have been an interesting approach to complete the study. Unfortunately, the limited amount of tumor tissue material did not allow evaluation by cytogenetics for 9p24.1 alterations. The use of multiplexed chromogenic or fluorescent immunohistochemistry (not available for our study) would allow a more detailed characterization of cellular spatial interactions [4]. However, by two different techniques (fresh cells analysis with flow cytometry and tumor staining with tumor paired biopsies), we found concordant results supporting that microenvironment of anti-PD-1 immunotherapy-resistant Hodgkin lymphoma were depleted in CD8+ T lymphocytes and overexpressed LAG-3.

## 5. Conclusions

Our study suggests that anti-PD-1 immunotherapy-resistant Hodgkin lymphoma could correlate in tumor microenvironment with an immune depletion of CD8+ T lymphocytes and overexpression of LAG-3 on CD4+ helper T lymphocytes. Further investigations in larger subsets of patients are warranted.

## Figures and Tables

**Table 1:** Main clinical characteristics of patients exposed to anti-PD-1 with classical Hodgkin lymphoma included in the study.

Patient Characterisics	Patients Exposed to Anti-PD-1			
	#1	#2	#3	#4
Age, sex (at time of tumor sample)	35, female	70, male	43, male	16, female
Hodgkin subtype *	Classical HL, Nodular sclerosis	Classical HL, Nodular sclerosis	Classical HL, Nodular sclerosis	Classical HL, Nodular sclerosis
Number of systemic therapies prior samples	7	6	5	2
BV therapy prior sample	Yes	Yes	Yes	No
ASCT prior sample	Yes	Yes	Yes	No
Anti-PD-1 regimen given (and dose) prior sample	Pembro 200 mg Q3W IV	Pembro 200 mg Q3W IV	Pembro 200 mg Q3W IV	Pembro 200 mg Q3W IV
Best response obtained with anti-PD-1 and % of maximum tumor decrease obtained	PR, (−) 91%	PR, (−) 76%	SD, (+) 25%	PR, (−) 54%
Number of anti-PD-1 cycles until biopsy sample	52 (two years)	52 (two years)	52 (two years)	52 (two years)
Time from last anti-PD-1 cycle to biopsy, in months	0.5	10.0	1.5	6.0
Technique of biopsy sample (needle core or adenectomy)	Adenectomy	Adenectomy	Adenectomy	Needle core biopsy
Part study for investigation	Part A and part B	Part B	Part A and part B	Part A

BV: brentuximab vedotin. ASCT: autostem cell transplantation. Anti-PD-1: anti-programmed death 1. HL: Hodgkin lymphoma. PR: Partial response. SD: Stable disease. Part A of the study was paired tumor biopsy with immunochemistry; part B of the study was based on flow cytometry on fresh cells of tumor microenvironment. * According to WHO 2016 classification of lymphoid neoplasms.

**Table 2:** Main clinical characteristics of control patients not exposed to anti-PD-1 with classical Hodgkin lymphoma included in the study.

Patient Characterisics	Patients Not Exposed to Anti-PD-1 (Control Patients)			
	#5	#6	#7	#8
Age, sex (at time of tumor sample)	55, male	76, male	35, female	40, male
Hodgkin subtype *	Classical HL Nodular sclerosis	Classical HL, Nodular sclerosis	Classical HL, Nodular sclerosis	Classical HL, Nodular sclerosis
Number of systemic therapies prior samples	1 (ABVD chemotherapy)	0 (untreated)	0 (untreated)	0 (untreated)
BV therapy prior sample	No	No	No	No
ASCT prior sample	No	No	No	No
Technique of biopsy sample (needle core or adenectomy)	Needle core biopsy	Adenectomy	Adenectomy	Adenectomy
Part study for investigation	Part B	Part B	Part B	Part B

ABVD: Adriamycine, Beomycine, Vinblastine, Dacarbazine. BV: brentuximab vedotin. ASCT: autostem cell transplantation. HL: Hodgkin lymphoma. Part B of the study was based on flow cytometry on fresh cells of tumor microenvironment. * According to WHO 2016 classification of lymphoid neoplasms.

## Data Availability

The data presented in this study are available on request from the corresponding author.

## Supplementary Materials

The following are available online at https://www.mdpi.com/article/10.3390/cancers13215487/s1, Figure S1: Immune cells distribution in control and post anti-PD-1 immunotherapy Hodgkin lymphoma patients. a/Illustration of flow cytometry data for CD45+ cells. b/The floating bars represent the proportion of CD45+ cells of the 2 groups of patients. One group is composed of patients (Control, n = 4) who are naive of treatment and the other group (post anti-PD-1, n = 3) of patients who relapsed after anti-PD-1 agonist antibody. Data are represented with the median.
