# Supplementary material for: CD8+ T Lymphocytes Immune Depletion and LAG-3 Overexpression in Hodgkin Lymphoma Tumor Microenvironment Exposed to Anti-PD-1 Immunotherapy
Source: Cancers (Basel). 2021 Oct 31;13(21):5487. doi: 10.3390/cancers13215487 (PMC8582920; doi:10.3390/cancers13215487)

Figure S1. Immune cells distribution in control and post anti-PD-1 immunotherapy Hodgkin lymphoma patients. a/Illustration of flow cytometry data for CD45+ cells. b/The floating bars represent the proportion of CD45+ cells of the 2 groups of patients. One group is composed of patients (Control, n = 4) who are naive of treatment and the other group (post anti-PD-1, n = 3) of patients who relapsed after anti-PD-1 agonist antibody. Data are represented with the median.

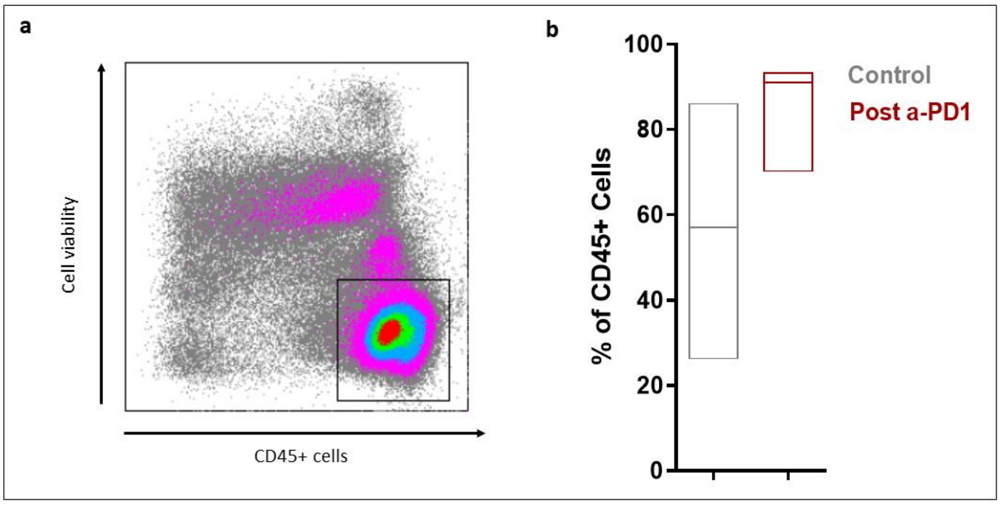

Supplement: Supplementary file 1 [file cancers-13-05487-s001.zip › cancers-1325513-supplementary.pdf]
